# Ten Thousand Dollars Reward

**Published:** 1858-06

**Authors:** 


### TEN THOUSAND DOLLARS REWARD

Is offered for a better receipt for Consumption than "*Dr. Hall's Balsam.*" Such is the heading of a handbill lately sent to us by mail. It goes on to state, that Dr. W. D. WRIGHT, Dr. J. S. LAWSON, Dr. WM. C. SMITH, and Dr. J. J. JOHNSON, all of Cincinnati, certify that it is entitled to the confidence of the public. It is for sale by A. L. SCOVILL & Co., No. 12 West Eighth Street, opposite the Methodist Book Concern, Cincinnati, Ohio. Our opinion is, that any man who uses it is a fool. We have been written to, to know if it was our preparation. We have known persons to die in the use of it, with the full assurance that it was our medicine. We have been abused in medical publications pretty roundly, for having anything to do with it. It was got up in Cincinnati, about ten years ago. Our name and reputation were stolen to give it a start, but the middle "W." left out, thus evading the law. We had no money to squander in chasing up a falsehood, nor the inclination to bother ourselves with setting the public to rights on the subject, said public being a sappy and irresponsible entity.

